# Supplementary material for: Chromothripsis during telomere crisis is independent of NHEJ, and consistent with a replicative origin
Source: Genome Res. 2019 May;29(5):737–49. doi: 10.1101/gr.240705.118 (PMC6499312; doi:10.1101/gr.240705.118)
Supplement: Supplemental Material [file supp_gr.240705.118_Supplemental_file_1.zip › contigs/annotated_contigs/DB89/contig.2.DB89_length_476_mean_cov_8.19117647059.docx]

**DB89_length_476_mean_cov_8.19117647059**

```
AAAATTATGCCATTATTTCAGGGGAAATATCTGAATTATGAAGTCACAGAAAGAAGATTATTTATATAGTTTTCAAGCACAATATAAAA
 >chr5:23359295-23359540 - E=6e-136 p=9e-03
CTGTTTTTTAATGTTGGGATCTATAATTTCATTAATGATGGAGAAAATGGGGCAGCATCACTCTTAATTTGCATTACACAAATGCACTT

ACATACTCTTTTTTTTTTTTCTAAATGGAGACTCTCTCTGTCACCAGGCTGGAGTGCAGTGGC|ATGA|CAAGATGCCCTCTTCTAATG
                                                                >chr5:23112218-23112453
ATATGCAAAATTCTCAGTCAACAAAGAATCCAAGCATCTTTTCTTTCTGATCAAAAAGAAACATGTATGACCTTACTGAACTAATATTT
 - E=7e-130
TACTGCACACTCTGAGTCCTGCCCAAACCCTCTTTTATTTTTCTTGAAGCAACTCGCATTATGTTTAATAATTTTTCTATATTTCATTT

AATTTATTCAAACTCACATGTGTTTAAGGTATA
```
